# Framework for types of metainferences in mixed methods research

**DOI:** 10.1186/s12874-025-02475-8

**Published:** 2025-01-24

**Authors:** Ahtisham Younas, Sergi Fàbregues, Sarah Munce, John W Creswell

**Affiliations:** 1 Memorial University of Newfoundland & Labrador, 300 Prince Philip Drive, St. John’s, Newfoundland and Labrador A1B 3V6 Canada; 2 Universitat Oberta de Catalunya, Barcelona, Spain; 3 Bloorview Research Institute, Holland Bloorview Kids Rehabilitation Hospital, Toronto, ON Canada; 4 University of Michigan, Ann Arbor, MI USA

**Keywords:** Mixed methods, Metainferences, Research methods, Data integration

## Abstract

**Background:**

The generation of metainferences is a core and significant feature of mixed methods research. In recent years, there has been some discussion in the literature about criteria for appraising the quality of metainferences, the processes for generating them, and the critical role that assessing the “fit” of quantitative and qualitative data and results plays in this generative process. However, little is known about the types of insights that emerge from generating metainferences. To address this gap, this paper conceptualize and present the types and forms of metainferences that can be generated in MMR studies for guiding future research projects.

**Methods:**

A critical review of literature sources was conducted, including peer-reviewed articles, book chapters, and research reports. We performed a non-systematic literature search in the Scopus, Web of Science, Ovid, and Google Scholar databases using general phrases such as “inferences in research”, “metainferences in mixed methods”, “inferences in mixed methods research”, and “inference types”. Additional searches included key methodological journals, such as the *Journal of Mixed Methods Research*, *International Journal of Multiple Research Approaches*, *Methodological Innovations*, and the *Sage Research Methods* database, to locate books, chapters, and peer-reviewed articles that discussed inferences and metainferences.

**Results:**

We propose two broad types of metainferences and five sub-types. The broad metainferences are global and specific, and the subtypes include relational, predictive, causal, comparative, and elaborative metainferences. Furthermore, we provide examples of each type of metainference from published mixed methods empirical studies.

**Conclusions:**

This paper contributes to the field of mixed methods research by expanding the knowledge about metainferences and offering a practical framework of types of metainferences for mixed methods researchers and educators. The proposed framework offers an approach to identifying and recognizing types of metainferences in mixed methods research and serves as an opportunity for future discussion on the nature, insights, and characteristic features of metainferences within this methodology. By proposing a foundation for metainferences, our framework advances this critical area of mixed methods research.

Integration is the cornerstone of mixed methods research (MMR) [1–3]. In MMR, integration can occur in 15 dimensions of the study, including the philosophical, theoretical, researcher, team; literature review, rationale, study purpose and research questions, data collection, data analysis, and interpretation dimensions, among others [3]. Of these dimensions, general consensus is that integration should occur at least at research design, question/purpose, data collection, analysis, interpretation, and reporting dimensions, with integration in the analysis and interpretation dimensions is arguably the most critical [1, 2, 4]. This form of integration involves the initial generation of individual inferences from the qualitative and quantitative strands, followed by the merging of the two to generate overall metainferences [2, 5, 6]. This process yields unique MMR insights that cannot be obtained using a monomethod approach. Metainferences are descriptions, conclusions, or insights generated at the end of an MMR study after careful analysis of the inferences drawn from the individual quantitative and qualitative strands (hence the term “metainferences”) and, more specifically, from the integration of the two databases [1, 2, 6]. Metainferences can take several forms, such as narratives, stories, or theoretical statements [7–9].

Although the generation of metainferences is a core feature of MMR [2, 5, 6], it remains an underdeveloped area within the field. Much has been written about the quality of metainferences, and some discussions on the process of generating them have been published [5, 6, 9–12]. To date, however, the MMR literature has not specifically addressed the types of metainferences that researchers can generate. Fetters [5] implicitly outlined several types of metainferences that researchers may draw when comparing the quantitative and qualitative data/results at the analysis and interpretation stages. However, comparing the two strands limits the perspectives on metainferences and does not probe alternatives that researchers might use. Given the challenges of forming metainferences, we need a typology of approaches that allows researchers to consider the full range of possibilities for drawing insights from the integration of quantitative and qualitative approaches [6]. Also, empirical MMR studies are needed to illustrate the various types of metainferences. By doing so, researchers will be able to see the different ways in which these types are used in practice and identify how to improve the processes and approaches related to integration (i.e., if we can name them, then we can study them, and if we can study them, we can iterate/improve them).

## Purpose

The purpose of this paper is to advance the types and conceptualize the forms of metainferences that can be generated in MMR studies for guiding future research projects. We explore the types of insights that emerge from the generation of metainferences by defining the concept, examining the available literature addressing the quality and rigor of metainferences and the process of generating them, and reviewing relevant sources. Finally, we propose five different types of insights that can be gained through the generation of metainferences.

### Definition of metainferences

In MMR, authors define metainferences in various ways, such as “negotiated account” [13], “narratives, or a story inferred from an integration of findings” [14], “process of theory development from observation” [14], “keystone” [7], “inferences that transcend both [qualitative and quantitative] databases” [15], “greater insights into a phenomenon and its implications” [16], and a “process of intensifying the mining of the data to gain a deeper and more accurate understanding of the phenomenon of interest” [6]. Drawing from the above-noted definition, in this paper, we define metainferences as overall conclusions drawn from the merging of qualitative and quantitative inferences that reveal unique insights which could not be achieved by either approach alone. Metainferences must be plausible, robust, and reflective of the data and inferences drawn from the individual strands of an MMR study [5, 6, 17].

### Metainferences in mixed methods research and the methodological gap

Discussions in the MMR field with respect to metainferences have followed two streams. Some MMR methodologists have discussed the quality and rigor of metainferences [9, 17–19] and proposed frameworks for critically appraising them. For example, Tashakkori and Teddlie [9] advanced an integrative framework for inference quality, such as interpretive rigor, which refers to the extent to which credible and plausible inferences and metainferences are drawn from raw and rigorously analyzed data. Interpretive rigor is comprised of five components: interpretive consistency, theoretical consistency, interpretive agreement, interpretive distinctiveness, and integrative efficacy. Onwuegbuzie and Johnson [18] offered a legitimation framework for assessing the validity of MMR studies. In their framework, they discussed integration legitimation, which assesses the quality of integration, and commensurability approximation legitimation, which involves conducting fully developed and rigorous qualitative and quantitative analyses. The discussion of these frameworks is beyond the scope of this paper, and researchers should refer to the sources and expanded discussions of these criteria [9, 18–20].

The second stream of discussion in MMR concerning metainferences focuses on both the processes to generate metainferences [6, 11, 21] and the types of metainferences that can be generated based on the consistency and inconsistency between the inferences drawn from each strand [5, 10]. For example, Schoonenboom [11] outlined a two-phase process for generating metainferences that entailed the generation of claims from individual qualitative and quantitative strands, followed by the compilation of these claims to generate metainferences for the overall study. Younas et al. [21] outlined two strategies, namely, comprehensive case analysis and sociocultural exploration, for generating and addressing discordant metainferences. Younas et al. [6] offered a seven-step process to generate metainferences, which included knowledge, experience, and data-driven inferences from each individual qualitative and quantitative strand, developing inference association maps to draw metainferences, and assessing the validity of metainferences using backward working heuristics.

Fetters et al. [4] and Fetters [5] reported that four types of metainferences can be generated in MMR. These include confirmed, discordant, expanded, and complementary metainferences. Confirmed metainferences are the result of identifying a consistency between the qualitative and quantitative inferences, while discordant metainferences reflect inconsistency between the two. Expanded and complementary metainferences are conceptualized as the partial or total divergence between inferences drawn from qualitative and quantitative strands [5]. Creswell [22] advanced a six-step simple model of the process of conducting MMR. In this model, metainferences are directly linked to integration and analysis using a joint display. Thus, researchers integrate the two databases in an MMR design, combine the databases through a joint display, and analyze the insights or metainferences gained by examining the joint display.

## Methods

This paper provides a critical review of literature sources, such as peer-reviewed articles, book chapters, and research texts on a particular topic, to offer a conceptual and theoretical understanding [23]. The end-products of critical reviews are theories, frameworks, and models [24]. We performed a non-systematic literature search in the Scopus, Web of Science, Ovid, and Google Scholar databases using general terms such as “metainference*”, “meta-inference*”, “inference* in research”, “metainference* in mixed method*”, “inference* in mixed methods research”, and “inference type*”. Additional search strategies included searching key methodological journals such as the *Journal of Mixed Methods Research*, *International Journal of Multiple Research Approaches*, *Methodological Innovations*, and the *Sage Research Methods* database to locate additional books, chapters, and peer-reviewed articles that discussed the topics of inferences and metainferences. Initial search retrieved 445 records after removing duplicates. Of these, 374 records were screened after reviewing their abstracts, titles, table of contents for books, and overview of chapters for book chapters. Finally, 71 methodological references were chosen which discussed inferences or metainferneces, their meaning, types and forms, and process of how metainferences are generated. We do not claim that this is a systematic methodological review. Because of the limited literature on metainferences, we selected literature sources using purposive sampling based on our reading and knowledge of the literature on inferences and metainferences in research, and our experience in designing and conducting MMR. We draw on a range of disciplines and fields —including social research methodology, MMR, teaching and learning of research methods, and philosophy of science— to generate a framework for various types of metainferences. Based on our previously published research on various MMR topics, such as prevalence reviews and reviews of joint displays and MMR interventions, we identify examples of empirical studies fitting under each type of metainference proposed in this framework.

### Framework for types of metainference insight

Our framework includes two broad types (global and specific) and five sub-types (relational, predictive, causal, comparative, and elaborative) of metainferences. These types are discussed and illustrated with examples from published MMR studies, as shown in Table 1.

**Table 1:** Characteristics and exemplars of types of metainferences

Types	Characteristics	Exemplars
Global	• Metainferences generated for populations and samples not directly studied in an MMR study. • Extrapolation of findings to draw metainferences for diverse populations with similar characteristics. • Metainferences generated based only on the information available in the qualitative and quantitative data. • These may appear as broad, generalized metainferences that can be translated across populations and contexts.	Karran et al. [75] conducted an MMR study using a convergent design to investigate individuals’ experiences of persistent pain and pain care, and to examine the consistency between clinical practice guidelines for managing pain and reported care practices. In the quantitative phase, individuals with spinal cord injury completed a survey, while in the qualitative phase, a nested sample participated in semi-structured interviews. A joint display was used for analysis and illustration for MMR integration. The authors provided general several metainferences that met the criteria of both global and specific. The global metainferences were clear in the sense that they could be extrapolated to other similar populations and contexts. Examples of global metainferences are as follows. • “Experiencing high levels of pain can impact self-efficacy, to the extent that the pain can ‘take over” [75] (p. 462). • “Self-monitoring and active self-management are key components of effective pain management” [75] (p. 462). • “Loss of personal relationships, the solitariness of their experience and the difficulty of finding a healthcare provider who understands contribute to a sense of loneliness and despair” [75] (p. 462).
Specific	• Metainferences generated for directly studied populations and samples. • Metainferences generated based only on the information available in the qualitative and quantitative data.	Younas, Porr et al. [69] conducted an exploratory sequential MMR study to understand the barriers to the delivery of compassionate care and to propose implementation strategies to promote compassionate nursing care for patients with multimorbidity and complex health needs. They identified patient perceived barriers and strategies to the delivery of compassionate care in the qualitative phase. In the integration phase, they used the *building* integration procedure incorporating qualitative data about barriers and three implementation sciences frameworks and theories to develop a Q survey entailing 21 implementation strategies to promote compassion. Based on a Q sort survey of nurses, policymakers, health care administrators, and compassionate care experts, they identified five highest and five lowest ranked implementation strategies. Finally, they merged qualitative data from patients about strategies to promote compassion with the ranked implementation strategies and generated 10 specific metainferences in a joint display. We labeled these implementation strategies as specific because they were highly contextualized to the Canadian health care context, for a very specific patient population, and for specific barriers. Examples of their drawn metainferences are as follows: **Implementation Strategy**: “Organize clinician implementation team meetings to support providers and provide them opportunities to reflect on implementing strategies for compassionate care towards complex patients” [69] (p. 14). **Metainference**: “Patients, nurses, nurse managers, administrators, managers, and policymakers emphasized the need to offer opportunities and resources for reflective practice” [69] (p. 15) **Implementation Strategy**: “Provide ongoing consultation with stress experts or counselors to address nurse burnout and to promote selfcare” (p. 14). **Metainference**: “Nurses, nurse managers, policymakers, and participants stated that nurses should be provided with resources to manage burnout, stress, and challenges when caring for complex patients” [69] (p. 14).
**Sub-Types**		
Relational	• Metainferences generated through identifying the relationships and linkages across two or more inferences about specific cases or constructs or variables. • Metainferences generated based only on the information available in the qualitative and quantitative data.	Firestone et al. [76] conducted a convergent MMR study and examined how preservice special educators’ engagement in teacher study groups impacted their equity-centered practices. They recruited 60 preservice special educators from a fieldwork support course and collected quantitative and qualitative data over four academic semesters in six courses. Each teacher study group included four to five educators and consisted of the following four parts: participants reflection on the implementation during the previous week, instructor presentation of new content, participant collaboration for implementation of new content, and instructor review and questions. The Classroom Assessment Scoring System (CLASS) was used for quantitative data to quantify instruction across emotional, organizational, and instructional domains. Qualitative data included reflections, student work samples, and lesson plans. Quantitative scores across various domains of the CLASS tool were used to determine educators’ growth-in-practice trajectories, and qualitative data provided insights into the features of participation that affected teachers’ growth in practice. During the MMR integration, they performed several relational analyses, such as comparing the CLASS scores of teachers with increased scores across most dimensions with those with decreased scores. They performed iterative analyses, presenting aggregated findings through quantitative analyses and single-participant and small-case configurations through qualitative findings. They developed a joint display of each educator’s pre-post scores across all dimensions of CLASS and then compared these findings with the aggregated qualitative data. The final meta-inferences were drawn for each case, and they were then drawn again after comparing the qualitative and quantitative inferences of several cases in relation to each other. An example of their metainference based on a joint display comparing the attitudes of two teachers is as follows: • “Attitudes regarding student deficits and pedagogy (teacher vs. student-centered instruction) influenced growth in practice” [76] (p. 10). • They further explained this metainference which demonstrate its relational nature. “Teachers who attributed instructional challenges to student deficits tended to show less growth in practice, whereas those who framed student challenges as signals to refine instruction tended to grow more” [76] (p.9)
Predictive	• Metainferences generated to forecast or unfold tentative events, mechanisms, future observations, or patterns concerning the studied phenomenon. • Metainferences generated solely on the basis of the information available in the qualitative and quantitative data.	Whitley et al. [77] conducted an explanatory sequential MMR study to better explain the predictors of effective prehospital pain management in children. Their quantitative phase was a cross-sectional survey to identify the predictors of pain management in children. They used the *connecting* integration procedure to select a sample for the qualitative phase and the *building* integration procedure to develop an interview guide for qualitative data collection. After the qualitative analysis, they developed a joint display to integrate the quantitative predictors and used the qualitative inferences to provide more contextual information about each of the predictors. They generated six metainferences pertaining to six predictors: age, gender, living in high or low deprivation area, analgesic use, type of pain, and paramedic care. Their metainferences were based on the data available for both phases and were presented as predictions; hence we labeled those as predictive inferences. Examples of their metainferences are presented as follows: • “Younger children achieve more effective pain management than older children. This was perceived to be because younger children express more emotion, therefore, are easier to distract, and they live more in the moment than their older counterpart” [77] (p. 8). • “Children attended by paramedics achieve more effective pain management than those attended by EMTs. This was perceived to be because paramedics are older, more experienced, more confident, have a greater scope of practice, and spend more time on scene than EMTs” [77] (p. 8).
Causal	• Metainferences that demonstrate cause-effect relationships between one or more quantitative variables and qualitative constructs. • Metainferences generated solely on the basis of the information available in the qualitative and quantitative data.	Emary et al. [78] used an explanatory sequential MMR study and examined the association between receipt of chiropractic services and initiating a prescription for opioids among adult patients with noncancer spinal pain. In the quantitative phase, they conducted a retrospective cohort study of 945 patient records and in the qualitative phase they interviewed 14 patients and nine general practitioners to explain the findings. Using hazard ratio and regression analysis, they generated quantitative inferences indicating that the risk of initiating a prescription for opioids at one year after presentation was lower in chiropractic recipients than in nonrecipients; lower in patients who received chiropractic services within 30 days of their index visit. The qualitative data supported their findings, thus generating causal metainferences. Two examples are illustrated as follows: • “The risk of receiving opioids was 52% lower in chiropractic recipients vs nonrecipients. Patients who were referred by their GP/NP for chiropractic services at Langs may have been more resistant to taking opioids than patients who were not referred for chiropractic services. Access to chiropractic treatment also gave GPs/NPs another nonopioid pain management option” [78] (p. 244). • “The risk of receiving opioids was 71% lower in patients who received chiropractic services within 30 days of their index visit. When accessed as a first-line treatment option, chiropractic care may have helped to delay, and in some cases prevent, the prescription of opioids” [78] (p. 244).
Comparative	• Metainferences that simply offer an integrated account of the inferences drawn from each strand. • These metainferences do not reflect a higher order synthesis of qualitative and quantitative inferences. • Metainferences generated solely on the basis of the information available in the qualitative and quantitative data.	Enggaard et al. [79] evaluated the impact of a guided self-determination intervention among adolescents with co-existing ADHD and medical disorders on their self-management and care involvement. They used a convergent MMR design with parallel data collection. In the quantitative phase, they used three scales to measure support from nurses, support from parents, and self-management of patients, while in the qualitative phase, they conducted interviews about patients’ experiences of the intervention, its impact on nurse support, parent support, and on their self-management. They used the *merging* integration procedure and developed two joint displays to illustrate qualitative and quantitative inferences and metainferences for each variable and construct. We labeled their metainferences as descriptive because they simply reported what they found in the two datasets. The metainferences did not reflect higher order insights. Examples of these descriptive metainferences are as follows: • “The qualitative results expand on the higher Health Care Climate Questionnaire (HCCQ) levels over time by highlighting the importance of content change and involvement” [79] (p. 93). • Perception of Parents Scale (POPS) scores were high throughout the intervention, confirming the finding that adolescents did not experience parental change.” [79] (p. 93).
Elaborative	• Metainferences generated to offer new and latent insights into the studied phenomenon. • Metainferences generated by going beyond the apparent meaning of the qualitative and quantitative inferences. • These metainferences can be generated by interpreting and drawing inferences beyond the information available in the qualitative and quantitative data.	Karran et al. [80] used a convergent MMR design to comprehensively understand the role of the social determinants of health in the care experiences and health status of socio-economically disadvantaged adults with persistent low back pain or persistent pain after a spinal cord injury. The quantitative phase included an online survey, and the qualitative phase included semi-structured interviews. After analyzing the qualitative and quantitative data separately, the authors used a joint display to illustrate the integration. They generated five metainferences to capture the pain care experiences of adults with low back pain and those with spinal cord injury and persistent pain. The joint display presented quantitative inferences, qualitative inferences, and complementary findings. We labeled the metainferences they drew as elaborative because they reflected a deeper interpretation of the data and went beyond the mere presentation of complementarity of results, and the metainferences can be applied to wider populations across contexts. Examples of their metainferences are as follows: • “Valued, trusting relationships impact perceived quality of care and health outcomes” [80] (p. 1475). • “Associations between social disadvantage and social isolation and their links with adverse outcomes reflect complex interrelationships between social determinants of health, access to quality care and pain-related outcomes” [80] (p. 1475).

### Broad types of metainferences

#### Global metainferences

Researchers can draw insights beyond the specific MMR study for other samples and populations. We define *global metainferences* as those generated to inform research, practice, and policy implications beyond the samples and populations studied in a specific MMR study. We have borrowed and adapted this term from the reading literature, where global inferences refer to those generated after comprehending the written texts or notes, but also drawing on background knowledge and global vocabulary (i.e., commonly used terminology in the field or subject being researched) [25–28]. In research, the text or notes are equivalent to the data gathered from participants at different phases of the study. In MMR, researchers can use metainferences to make claims and generalizations about populations and phenomena [29–31], or they can use them to make analytical generalizations, i.e., to generalize the findings to a broader theory or model to support their assumptions and premises about a specific phenomenon [31, 32]. When global metainferences are generated in an MMR study, researchers and readers of the study have the opportunity to extrapolate these metainferences to inform practice and policy in their relevant contexts and settings.

#### Specific metainferences

Researchers can draw insights from the data, the sample, and the population studied in an MMR study. We define *specific metainferences* as those that are directly relevant to the sample and population studied in an MMR study. In the reading literature, the term “local inference” is used to refer to those inferences and conclusions that are made based on the available information and texts without incorporating background knowledge and global vocabulary [25–28]. We refer to these types of metainferences as “specific”. All MMR studies make knowledge, practice, and policy claims about the population and sample studied [5, 33]. Therefore, these metainferences capture those claims and conclusions that directly add to the knowledge about the studied phenomenon in the specific population and potentially inform future research and practice. Under global and specific metainferences, researchers can generate any of five subtypes of metainferences, namely, relational, predictive, causal, comparative, and elaborative. These subtypes are explained below.

### Sub-types of metainferences

#### Relational metainferences

Researchers can draw theoretical insights about from metainferences. We define *relational metainferences* as those that are generated through identifying the relationships and linkages across two or more inferences about specific cases, constructs, or variables. Thus, the researcher converts the inferences from qualitative and quantitative strands into higher order metainferences that are relative to each other and interdependent. In the research methods and logic literature, authors discuss relational inferences as interpretations drawn after examining correlations [34–36] and assessing the analogical connection [36, 37] among variables or constructs. Thus, authors generate relational inferences after examining conceptual or semantic connections between two or more statements and inferring their shared meaning [28]. In MMR, researchers can draw relational metainferences during case-by-case analysis [12] or by undertaking a comprehensive case analysis at the level of a single quantitative variable or qualitative construct [21]. In either case, relational analyses in MMR enable researchers to examine the linkage and contingency of one dataset with another [1]. Therefore, relational metainferences represent an advanced level of metainferences, as they go beyond mere descriptions by integrating qualitative and quantitative inferences. As Fetters [5] argued, generating higher-order metainferences in advanced MMR analysis consists of examining, exploring, and moving beyond the apparent relationships of data and inferences to highly complex and extensive interpretations and theory development. Therefore, relational metainferences can enable theory generation in MMR.

#### Predictive metainferences

Researchers can draw predictive insights from metainferences. *Predictive metainferences* are those that forecast or unfold tentative events, mechanisms, future observations, or patterns concerning the studied phenomenon in MMR studies. In probability theory [38, 39], predictions can be categorized as statistical and clinical inferences, the latter being based on clinicians’ experiences in practice and their knowledge and understanding of the data [40]. In the reading literature, predictive inferences are the expectations that a reader can make about the text that has not yet been read; therefore, it requires qualified judgments to forecast future events [28, 41]. Similarly, in MMR, predictive metainferences can be drawn through careful examination of data and validation of inferences from individual strands with qualified judgments about the studied phenomenon. Predictive metainferences can be foundational in generating areas for future research.

#### Causal metainferences

Researchers can draw causal insights from metainferences. We define *causal metainferences* as those demonstrating a cause-effect relationship based on the qualitative and quantitative inferences about qualitative findings and quantitative data. Causal metainferences illustrate a causal relationship drawn from metainferences, while relational metainferences demonstrate a relationship between variables or constructs. Causal metainferences differ from predictive inferences in that the qualitative data offers contextual, rich, and thick information to strengthen predictions. In the quantitative methods literature, causal inferences are commonly discussed in terms of statistical inference [42, 43], which is based on the counterfactual model, which posits that in the absence of a necessary causative factor, a certain outcome cannot occur [44]. Causal inferences can also be time-, context-, and population-specific [45], and are usually generated based on powerful and robust statistical testing and modeling analyses [42, 43, 45, 46]. Causal inference in qualitative research is also plausible because qualitative data offer a detailed description of the context [46, 47], can help identify the conditions under which certain causal relationships work, can provide in-depth information about the mechanism of causal relationships, and can offer theoretical accounts of causality [48–53]. Johnson et al. [54] argued that causation in qualitative research are local causation inferences that can be drawn in qualitative studies as they help to generate insights into complex processes and mechanisms of causation. Causal inferences can be generated in MMR as demonstrated by Johnson et al. [54] in their pluralistic causal theory. Bazeley [1] noted that the causal inferences are generated in MMR by using quantitative data to answer the “what” and qualitative data to answer the “how, why, and to what extent” of causal associations. Therefore, since causal inferences are drawn from individual qualitative and quantitative strands of an MMR study, the distinct inferences can be integrated to generate causal metainferences.

#### Comparative metainferences

Researchers can draw integrative inferences from each strand of an MMR study. We define *comparative metainferences* as those that simply offer an integrated account of the inferences. These types of metainferences do not require higher-order synthesis or latent synthesis and are more surface level or semantic. Comparative insight represents the type of metainference that was advanced by Fetters [5] when he discussed the “fit” between the quantitative and qualitative databases. He noted that this “fit” could be one of confirmation, discordance, expansion, and complementarity. In the research literature, descriptive inferences merely aim to offer answers to unobserved entities in terms of what, who, when, where and why [55] and summarize the data to draw summary interpretations about the phenomenon or different aspects of the phenomenon [56, 57]. Comparative or descriptive inferences are limited because they should be generated based on the available set and nature of observations [56, 58]. Therefore, comparative metainferences in MMR can simply be straightforward explanations of the data and findings and may serve as potential candidates for informing future research on the specific phenomenon. As Bazeley [1] noted, when writing up MMR results, researchers sometimes only need only to describe their insights, and they do so by identifying building blocks, key insights, or patterns of association and synthesizing them into a descriptive account.

#### Elaborative metainferences

Researchers can elaborate on the quantitative and qualitative inferences. We define *elaborative metainferences* as those offering new and latent insights generated from the integration of qualitative and quantitative inferences in an MMR study with careful consideration of the available contextual information. In the reading literature, elaborative inferences are drawn interpretations about associations and characteristics of text by moving beyond what is apparent in the text and embellishing details of the text by incorporating contextual information retained in one’s memory about the studied phenomenon [28, 41, 59]. The elaboration of hidden phenomena or their latent aspects is one of the key features of MMR research, because a meaningful combination of qualitative and quantitative data enables the unraveling of unique phenomena and the drawing of insightful inferences [5, 21, 60]. These elaborations may also allow for proximal theory building [60]. Hence, elaborative metainferences are conceptualized as latent and unique insights drawn from the careful integration and combination of qualitative and quantitative inferences by expanding one’s interpretation beyond the apparent meanings. Nevertheless, these metainferences are still rooted in the data.

## Discussion

Several prevalence reviews of MMR have demonstrated a growing use of MMR across the health [61–64] and social and behavioral sciences [65–68] to study a diverse range of phenomena. The growing use of MMR across these disciplines indicates that clinicians, educators, managers, social and behavioral scientists, readers of research, and policy makers rely on the use of evidence from MMR to inform research, practice, and policy [5, 69]. Therefore, the insight, interpretations, conclusions, and recommendations generated from MMR studies need to be relevant, accessible, and meaningful to meet the needs of these diverse groups. These insights, one could say, represent the value of undertaking an MMR study. Furthermore, drawing them is a significant feature of MMR, and authors refer to them as “metainferences” [1, 5, 11]. Unfortunately, we know little about these metainferences beyond the need for them to be of high quality [9], the steps involved in generating them [6], and the results of comparing the quantitative and qualitative databases [5]. What insights can researchers gain from metainferences? To answer this question, we have presented six approaches organized into global and specific approaches. Researchers can draw insights for samples and populations beyond the specific MMR study, theory development, prediction, causal relationships, comparisons, and/or elaborations. Furthermore, we advance empirical MMR published studies to illustrate each of our types. All the metainference types in our framework are useful to understand factors affecting health and well-being providing clear, relevant, and high-quality conclusions. Metainferences offer readers the opportunity to evaluate the relevance of their use in their own research and practice [31, 70].

While we offered various types of metainferences, not all types may be applicable to all MMR studies. The generation of metainferences may be contingent on the purpose, design, and objectives of MMR studies. Nevertheless, when researchers analyze data and generate inferences from the integration, metainferences are drawn directly from the data after interpretation of inferences from each phase [5, 71]. Therefore, a framework of various types of metainferences serves as a foundation for choosing relevant types of metainferences to better articulate and label additional insights gained during analysis. Another potential advantage of generating varied types of metainferences is optimizing the potential of MMR to help generate new predictive, descriptive, and causal theories [14, 54, 72].

Being able to identify the type of metainferences enables researchers to write up and present metainferences using visual forms. For example, researchers may present causal, relational, and predictive metainferences as equations, figures, and theoretical models. While authors often discuss metainferences in various types of tabular and visual joint displays in separate columns [73, 74], the use of tabular and graphical modes to illustrate the juxtaposition of multiple types of metainferences drawn about one phenomenon can add greater value, depth, and analytical insight. Bazeley [1] emphasized that the use of visual modes as tools for data analysis, as well as for communicating interpretations and conclusions, allows for a better understanding of the implications of MMR findings.

### Contribution to research methodology

This paper contributes to the field of MMR by expanding the discussion by providing types of insights emerging from metainferences. As such, it offers a practical tool for MMR researchers and educators. The types of metainferences proposed in this paper can guide and inform new innovative methods to generate each type within specialized MMR designs. For example, if a researcher is interested in drawing causal metainferences, then perhaps the metainference generation process may entail giving greater weight to the quantitative inferences and using qualitative inferences to strengthen the generated metainferences. Building on the proposed types of metainferences, MMR researchers can design and develop more advanced approaches to generating metainferences.

The proposed framework can be a useful teaching tool for educators teaching MMR, as it can allow novice researchers to become cognizant of the types of metainferences relevant to the purposes of their MMR studies. Instructors can educate learners about the different types of metainferences to promote integrative thinking for better design of their MMR studies. If MMR researchers identify the types of metainferences that they intend to draw from their MMR at the conceptualization stage, they can hone the emergent design approaches to ensure that high-quality data are collected during the study.

## Conclusions

The MMR literature shows tremendous growth in terms of integration methods, approaches, design typologies, and quality criteria. However, limited guidance exists concerning the types of insights or conclusions that can be drawn from metainferences. There is a consensus in the MMR literature that metainferences are an essential output in MMR studies. Therefore, knowledge of how different types of metainferences can be drawn enables researchers to recognize the type informing their analytical choices. Our proposed framework offers one approach to identifying and recognizing the types of metainferences in MMR and serves as an opportunity for future discussion of the nature, types, insights, and characteristic features of metainferences in MMR. Our framework suggests a foundation for metainferences and advances this critical area of MMR.

## Data Availability

No datasets were generated or analysed during the current study.
